# Efficacy of the mRNA-Based BNT162b2 COVID-19 Vaccine in Patients with Solid Malignancies Treated with Anti-Neoplastic Drugs

**DOI:** 10.3390/cancers13164191

**Published:** 2021-08-20

**Authors:** Abed Agbarya, Ina Sarel, Tomer Ziv-Baran, Sivan Agranat, Orna Schwartz, Ayelet Shai, Sharon Nordheimer, Shlomit Fenig, Yelena Shechtman, Ella Kozlener, Tarek Taha, Haitam Nasrallah, Roma Parikh, Nadav Elkoshi, Carmit Levy, Rasha Khoury, Ronen Brenner

**Affiliations:** 1 Bnai-Zion Medical Center, Oncology Institute, 47 Golomb Avenue, Haifa 3339419, Israel; Yelena.Shechtman@b-zion.org.il (Y.S.); Ella.Kozlener@b-zion.org.il (E.K.); rasha.khoury@b-zion.org.il (R.K.); 2 Edith Wolfson Medical Center, Oncology Institute, 62 Halohamim Street, Holon 5822012, Israel; Inasa@wmc.gov.il (I.S.); SivanA@wmc.gov.il (S.A.); sharonno@wmc.gov.il (S.N.); shlomitf@wmc.gov.il (S.F.); 3 School of Public Health, Sackler Faculty of Medicine, Tel-Aviv University, 35 Klatchkin Street, Tel-Aviv 6997801, Israel; Zivtome@tauex.tau.ac.il; 4 Microbiology and Immunology Laboratory, Edith Wolfson Medical Center, 62 Halohamim Street, Holon 5822012, Israel; OrnaS@wmc.gov.il; 5 Department of Oncology, Galilee Medical Center, P.O. Box 21, Nahariya 22100, Israel; AyeletS@gmc.gov.il; 6 Department of Oncology, Rambam Health Care Campus, 8 HaAlyia Hashniya, Haifa 31096, Israel; t_taha@rmc.gov.il (T.T.); h_nasrallah@rmc.gov.il (H.N.); 7 Department of Human Genetics and Biochemistry, Sackler Faculty of Medicine, Tel-Aviv University, Tel-Aviv 69978, Israel; romaparikh@live.in (R.P.); nadav.elkoshi@gmail.com (N.E.); carmitlevy@post.tau.ac.il (C.L.)

**Keywords:** cancer, chemotherapy, immunotherapy, COVID-19 vaccine, SARS-CoV-2, antibody response

## Abstract

**Simple Summary:**

Cancer patients undergoing active treatment may be more vulnerable to COVID-19 due to the effects of the malignant disease and the anti-cancer treatment. Assessing COVID-19 vaccine efficacy in protecting these patients from SARS-CoV-2 infection, is important due to the worldwide pandemic reaching an unprecedented third surge. Cancer patients on active neoplastic treatment had a reduced antibody response following immunization with the BNT162b2 vaccine, manifested as significantly more seronegative results and lower antibody levels compared with those of non-cancer individuals. The risk of a negative serological response was 7.35 times higher in patients on active anticancer treatment. This impaired serological response was mostly attributed to chemotherapy treatment. Cancer patients receiving non-chemotherapy treatments had a similar serologic response as those without cancer. Vaccinated cancer patients treated with chemotherapy might not be protected against SARS-CoV-2 infection and may need to continue exercising precautionary measures such as social distancing and facial masking.

**Abstract:**

The BNT162b2 vaccine was shown to be highly effective in reducing the risk of COVID-19 infection in healthy individuals and patients with chronic disease. However, there are little data regarding its efficacy in patients treated for cancer. We analyzed the humoral response following vaccination with the second dose of BNT162b2 in 140 patients with solid malignancies who were receiving anti-cancer therapy at the time of vaccination and 215 participants who had not been diagnosed with cancer. Multivariate analysis was performed, followed by matching the two groups by age, gender and days from vaccination. The humoral response in the cancer patient group was significantly lower than in the non-cancer group: 20/140 seronegative (14.3%) vs. 3/215 (1.4%), *p* < 0.001; median IgG levels 2231 AU/mL (IQR 445-8023) vs. 4100 (IQR 2231-6774) *p* = 0.001 respectively. The odds ratio for negative serology results in cancer patients adjusted by age and gender was 7.35 compared to participants without cancer. This effect was observed only in chemotherapy treated patients: 17/73 seronegative (23.3%) vs. 3/215 (1.4%), *p* < 0.001; median IgG 1361 AU/mL vs. 4100, *p* < 0.001 but not in patients treated with non-chemotherapeutic drugs. Reduced immunogenicity to COVID-19 vaccine among chemotherapy-treated cancer patients, raises the need to continue exercising protective measures after vaccination in these patients.

## 1. Introduction

Coronavirus disease 2019 (COVID-19), caused by the Severe Acute Respiratory Syndrome Coronavirus 2 (SARS-CoV-2) infection, has currently affected more than 147 million patients worldwide, leading to more than three million deaths [1]. This pandemic has affected almost all aspects of medical care, including cancer care [2]. Patients with cancer are at increased risk for COVID-19 complications and mortality, most likely due to the effects of the underlying malignancy and immunosuppressive treatments, thus effective preventive measures for this population are urgently required [3,4,5].

The lipid nanoparticle-formulated, chemically modified RNA vaccine, BNT162b2 (Pfizer-BioNTech), has an acceptable safety profile and had a 95% efficacy rate in preventing COVID-19, regardless of age, sex, race, ethnicity, or baseline body-mass index in clinical testing [6,7]. The clinical trial data submitted to FDA for the approval of BNT162b2 included data on eligible volunteers who were medically healthy or who had chronic conditions. Patients undergoing treatment with immunosuppressive therapy, including anti-cancer therapy, were excluded from the clinical trials [7].

It is agreed that cancer patients who are receiving anti-neoplastic treatments should be prioritized for vaccination [8,9]. However, data regarding safety and efficacy of the BNT162b2 mRNA COVID-19 vaccine in this group of patients are lacking [10,11]. The ideal timing of vaccination relative to anti-neoplastic therapy is yet to be determined. The Centers for Disease Control and Prevention (CDC) recommends that vaccination be delivered 2 weeks prior to immunosuppressive therapy [12], but this is not possible in patients already on chemotherapy [12,13]. Several new studies raised concerns about the blunted antibody responses to the COVID-19 vaccine in patients with hematological malignancies [14,15,16]. A recent study reported that the BNT162b2 vaccine does have an acceptable short-term safety profile in patients treated with immune checkpoint inhibitors [17], but data on the efficacy of the vaccine in cancer patients with solid malignancies treated with anti-neoplastic drugs are scarce [18,19].

On December 2020, the Israeli Ministry of Health (MOH) launched a national vaccination program for individuals 16 years of age and older [20]. A high percentage of the population was vaccinated in a relatively short time [21,22]. Patients with chronic diseases and cancer were prioritized to be vaccinated and cancer patients at our institutions were encouraged to receive the vaccine. The aim of the present study was to assess the serological response to the BNT162b2 vaccine among cancer patients. The study is essential to evaluate the ability of this vulnerable population to mount an effective humoral response, while on active anti-neoplastic treatment during the COVID-19 pandemic.

## 2. Materials and Methods

### 2.1. Study Design and Partipants

This cross-sectional study was carried out at the Edith Wolfson Medical Center (WMC) and Bnai Zion Medical Center (BZMC) between 22 February and 8 April 2021. Two groups were included: cancer patients with solid tumors who were undergoing active anti-cancer treatment when the first and second doses of the BNT162b2 vaccine were administered and a contol group of vaccinated individuals who did not have cancer. All enrolled participants were at least 18 years old and have received both doses of the BNT162b2 vaccine between January and March 2021 according to the manufacturer’s (Pfizer-BioNTech, Kalamazoo, MI, USA) instructions. The participants were asked about age, gender, previous cancer disease, history of exposure to COVID-19 and vaccination dates. Clinical data regarding cancer types, treatment regimens and protocols were obtained from the medical charts.

Patient characteristics are summarized in Table 1. Patients included in the study had gastrointestinal cancers (colon, gastric, esophagus, ampullary, pancreas and cholangiocarcinoma), breast, lung, urinary cancers (bladder, kidney, and prostate), gynecological cancers (ovarian and uterine), melanoma, sarcoma, and head and neck cancer. One patient had non active chronic lymphocytic leukemia in addition to a solid tumor. Patients undergoing treatment were analyzed based on treatment type, chemotherapy versus non-chemotherapy (immunotherapy and biological drugs), and according to whether treatment was metastatic, adjuvant, or neoadjuvant. Chemotherapy drugs used included: gemcitabine, eribulin, paclitaxel, carboplatin, oxaliplatin, cisplatin, irinotecan, fluorouracil, etoposide, pemetrexed, vincristine, doxorubicin, and cyclophosphamide. Immunotherapy drugs included pembrolizumab, nivolumab, ipilimumab, durvalumab, avelumab, atezolizumab, and cemiplimab. Biological drugs included trastuzumab, pertuzumab, panitumumab, ribociclib, axitinib, regorafenib, olaparib, and lenvatinib.

The comparison group of subjects who did not have cancer included patients’ relatives, health-care workers, and volunteers in our medical centers. Individuals in this group had also received both doses of the BNT162b2 vaccine. None of the subjects in the comparison group had a previous cancer diagnosis. Chronic steroid treatment and reported evidence of SARS-CoV-2 infection were exclusion criteria for both groups. The study was approved by the Institutional Review Boards of both medical centers (protocols 0010-21 WOMC and 0009-21-BNZ, respectively). All participants signed a written informed consent.

### 2.2. Assessment of Anti-SARS-CoV-2 Antibodies

Peripheral venous blood samples of 6–8 mL were obtained from the participants at least 7 days after the administration of the second vaccine dose. Serum was collected and stored at 2 to 8 °C for a maximum of 7 days prior to analysis. IgG testing was performed at the WMC Immunology Laboratory using the SARS-CoV-2 IgG II Quant assay on an ARCHITECT analyzer (Abbott). This assay, which has received authorization from the Israel MOH and the US Food and Drug Administration, is a high-throughput chemiluminescent microparticle immunoassay designed to quantitatively measure IgG antibodies that bind to the receptor binding domain of the S1 subunit of the SARS-CoV-2 spike protein. The assay has a measurement range of 6.8–40,000 arbitrary units (AU) per ml. Antibody levels lower than 50.0 AU/mL are considered negative, whereas values greater than 150 AU/mL are regarded as positive. Values between 50–150 AU/mL are defined by manufacturer as borderline. For the purpose of the analysis, participants with borderline response were considered negative.

### 2.3. Statistical Analysis

Categorical variables were summarized as frequency and percentage. Continuous variables were evaluated for normal distribution using histogram and Q-Q plot and reported as Mean and Standard Deviation (SD) for normally distributed variables or as Median and Interquartile Range (IQR) for skewed variables. χ^2^ Test or Fisher Exact Test were used to compare between groups. Continuous variables were compared using Independent Samples *t* Test or the Mann–Whitney test. Crude and Adjusted Odds Ratio were evaluated using logistic regression. Age, gender, days from vaccination were included in the multivariate analysis.

In further analysis, the two groups were matched according to age (±3 years), gender and days from vaccination (within 14 or 7 days of each other). Paired samples’ *t* test and Wilcoxon Test were used to compare continuous variables between the matched groups, while McNemar test was applied to compare categorical variables. All statistical tests were two tailed. *p* < 0.05 was considered statistically significant. All statistical analyses were performed using SPSS (IBM SPSS Statistics for Windows, version 24, IBM Corp., Armonk, NY, USA, 2016).

## 3. Results

### 3.1. Study Population

A total of 290 cancer patients with solid tumors were treated during February and March 2021 at both medical centers. Of them, 183 patients had received at that time both doses of the BTN162b2 mRNA vaccine and were examined for eligibility. In total, 43 patients did not meet the primary inclusion criteria of receiving active treatment around the vaccination time. The final analysis included 140 cancer patients with solid tumors, receiving anti-neoplastic treatments and a comparison group of 215 participants without cancer. The participants’ characteristics and the cancer types are summarized in Table 1. The most frequent cancer types were gastrointestinal (48 (34.2%)), breast (30 (21.4%)) and lung (27 (19.3%)). The great majority of patients had metastatic disease (109 (77.8%)). Treatment protocols were chemotherapy (75 (52%)) and non-chemotherapy drugs consisting mostly of immunotherapy (43 (65%)) and biological drugs. The analysis of the response to BTN162b2 according to tumor type is presented in Table S1. The results stratified by three IgG antibodies categories (Negative: <50 AU/mL, Borderline: 50–150 AU/mL and Positive: >150 AU/mL) are summarized in Table S2.

### 3.2. Response to BNT162b2 in Patients with Cancer versus Participants without A Cancer Diagnosis

**Figure 1:**
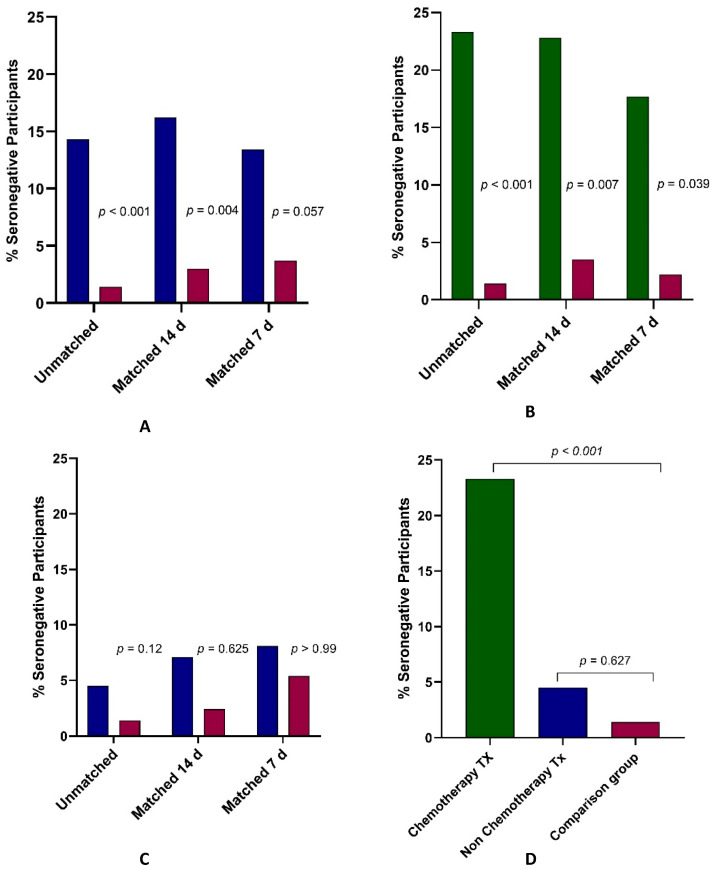
Vaccine response: (**A**) percent of seronegative patients with cancer (blue bars) and non-cancer participants (maroon bars) in unmatched analysis (cancer patients, *n* = 140; controls, *n* = 215, *p* < 0.001) and matched analyses (±14 days following second vaccine dose, *n* = 99 pairs, *p* = 0.004; and ±7 days following second vaccine dose, *n* = 82 pairs, *p* = 0.057); (**B**) percent of seronegative cancer patients undergoing chemotherapy at the time of vaccination (green bars) compared to non-cancer participants (maroon bars) in unmatched analysis (cancer patients, *n* = 73; controls, *n* = 215, *p* < 0.001) and matched analyses (±14 days, *n* = 57 pairs, *p* = 0.007; and ±7 days, *n* = 45 pairs, *p* = 0.039); (**C**) percent of seronegative cancer patients undergoing non-chemotherapy treatment at the time of vaccination (blue bars) compared to controls (maroon bars) in unmatched analysis (cancer patients, *n* = 67; controls, *n* = 215) and matched analyses (±14 days, *n* = 42 pairs; and ±7 days, *n* = 37 pairs). In all analyses, the differences were not significant (NS); (**D**) in the unmatched analysis, percent of seronegative participants in the subgroups of patients undergoing chemotherapy treatment (Tx) (*n* = 73), non-chemotherapy treatment (*n* = 67), and comparison group (*n* = 215).

**Figure 2:**
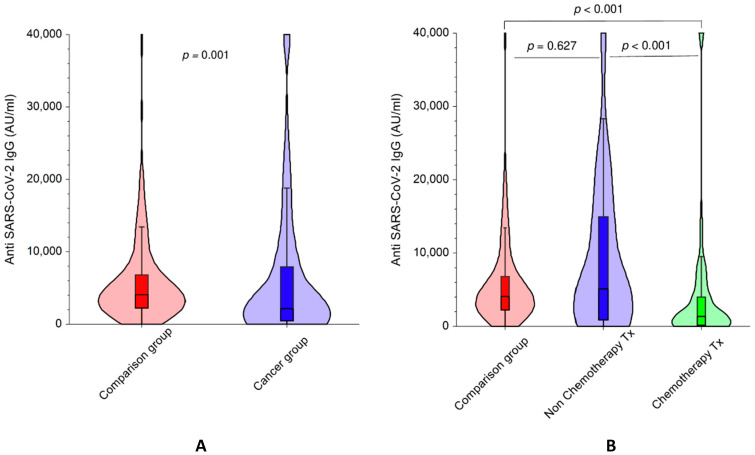
SARS-CoV-2 IgG antibodies titer. Violin graphs and box plots of the distribution of anti-SARS-CoV-2 antibody levels (AU/mL). The median and quartiles are presented as horizontal lines within the box plot. (**A**) Cancer and comparison non-cancer groups (unmatched). The median SARS-CoV-2 IgG levels, (2231 AU/mL) were significantly lower in the cancer group than in the comparison group, median 4100 AU/mL, (*p* = 0.001). The lower IgG values are more frequent in the cancer group; (**B**) chemotherapy-treated, non-chemotherapy-treated and comparison groups (unmatched). The lower IgG values, as apparent from the wider bottom distribution, are more frequent in the chemotherapy treated group. The antibody levels in the chemotherapy group, median 1361 AU/mL, but not in the non-chemotherapy group, median 5088 AU/mL, are significantly lower than the comparison group, median 4100 AU/mL, *p* < 0.001 and *p* = 0.627 respectively. Median immunoglobulin levels, represented by the middle horizontal lines, were significantly lower in the chemotherapy-treated patients: 1361 AU/mL versus 5088 AU/mL in non-chemotherapy treated patients (*p* < 0.001).

A total of 20 (14.3%) of the 140 cancer patients did not develop antibodies against the S1 subunit compared to only 3 (1.4%) of 215 the non-cancer participants (*p* < 0.001) (Figure 1A). The median SARS-CoV-2 IgG levels were also significantly lower (*p* = 0.001) in cancer patients, 2231 AU/mL (IQR 445-8023) than in the control group of subjects without a cancer diagnosis, median 4100 AU/mL (IQR 2231-6774) (Figure 2A; Table 2). In a multivariate logistic regression analysis including age, gender, and days from the second vaccine dose, the odds ratio (OR) of cancer patients not developing SARS-CoV-2 IgG antibodies was 7.35 compared to participants without cancer (95% confidence interval (CI) 1.9–27.8); *p* = 0.003.

We next conducted analyses with cancer patients and subjects who had not been diagnosed with cancer matched according to age (±3 years), gender, and vaccination within 14 days of each other, resulting in 99 matched pairs. There were differences between the groups in the humoral antibody response: 16 (16.2%) of 99 cancer patients did not develop SARS-CoV-2 IgG antibodies compared to three (3%) of 99 in the matched control group (*p* = 0.004) (Figure 1A). The median levels of immunoglobulins did not differ significantly even though the IQRs were very different: 1771 AU/mL (IQR 284-7292) in cancer patients compared to 2993 AU/mL (IQR 1775-6002) in the control group (*p* = 0.52). The first quartile (Q1) values in particular were much lower in cancer patients: 284 AU/mL versus 1775 AU/mL (Table 2).

### 3.3. Response to BNT162b2 in Cancer Patients Receiving Chemotherapy versus Participants without Cancer

Since the humoral immune response might be related to the type of anti-neoplastic treatment, we performed a further analysis based on treatment type. The cancer group was divided into two subgroups: patients who were undergoing chemotherapy at the time of vaccination and patients receiving non-chemotherapy regimens. The group of 73 patients who were being treated with chemotherapy was compared with the control group of 215 participants. Of the 73 cancer patients on chemotherapy, 17 (23.3%) were seronegative compared to three (1.4%) of 215 in the non-cancer group (*p* < 0.001). (Figure 1B). Median immunoglobulin levels were significantly lower in the chemotherapy-treated patients: 1361 AU/mL versus 4100 AU/mL for controls (*p* < 0.001) (Figure 2B; Table 2).

The reduced humoral antibody response among the chemotherapy treated patients was also evident in matched groups. Of 57 pairs of chemotherapy-treated cancer patients and controls with an interval between vaccination and serology blood sampling within 14 days, 13 (22.8%) of 57 cancer patients receiving chemotherapy did not have detectable antibodies against the SARS-CoV-2 virus compared to two (3.5%) of 57 non-cancer participants (*p* = 0.007) (Figure 1B). The patients undergoing chemotherapy treatment also had significantly lower median levels of immunoglobulins than matched controls (821 AU/mL vs. 3559 AU/mL, *p* = 0.035) (Table 2).

Analysis of the groups matched within 7 days of each other from vaccination revealed similar results. Eight (17.7%) of the 45 cancer patients had no SARS-CoV-2 IgG antibodies above threshold, compared to only one (2.2%) of 45 individuals in the matched control group (*p* = 0.039) (Figure 1B). The median immunoglobulin levels in the chemotherapy-treated cancer patients were significantly lower than those of the matched control participants (864 AU/mL vs. 3329 AU/mL, *p* = 0.013) (Table 2).

### 3.4. Response to BNT162b2 in Cancer Patients Receiving Non-Chemotherapy Regimens versus Participants without Cancer

Very different results were observed when the non-chemotherapy treated group, consisting of 67 cancer patients who received immunotherapy or a biologic treatment, was compared to the control group (Figure 1D). There were no significant differences between the groups in the percentage of seronegative participants: Three (4.5%) of 67 non-chemotherapy cancer patients and three (1.4%) of 215 in the control group (*p* = 0.12) were seronegative (Figure 1C). Likewise, no significant differences were demonstrated in the immunoglobulin median levels (5088 AU/mL in patients vs. 4100 AU/mL in controls, *p* = 0.627) (Figure 2B; Table 2). Similar results were obtained in the matched groups. Three (7.1%) of 42 cancer patients were seronegative versus one (2.4%) of 42 controls (*p* = 0.625) matched according to age (±3 years), gender, and within 14 days from vaccination (Figure 1C). In groups matched within 7 days of each other from vaccination, three (8.1%) of 37 cancer patients did not develop SARS-CoV-2 IgG antibodies above threshold compared to two (5.4%) of 37 controls (*p* > 0.99) (Figure 1C). The difference in IgG levels was not statistically significant difference between the two groups (Figure 2B).

The serological response in immunotherapy treated patients, the majority of patients receiving non-chemotherapy regimens, was also not significantly different compared to participants without cancer (Table 2, Figure S1).

### 3.5. Response to BNT162b2 in Cancer Patients Receiving Chemotherapy versus Non-Chemotherapy Regimens

Significantly more cancer patients receiving chemotherapy, 17 of 73 (23.3%) were seronegative compared to three (4.5%) of 67 cancer patients treated with non-chemotherapy drugs (*p* = 0.001) (Table 2 and Table S3). Median immunoglobulin levels were significantly lower in the chemotherapy-treated patients: 1361 AU/mL versus 5088 AU/mL in non-chemotherapy treated patients (*p* < 0.001) (Figure 2B; Table 2 and Table S2). There was no difference in the number of days from the second BNT162b2 vaccine dose between the groups (*p* = 0.66), (Table S3).

The Spearman correlation method was used to assess the correlation between the IgG values and the number of days after vaccination.

Overall, for all participants there was no significant correlation between the Ig G titer and the time from the second vaccination dose. However, after dividing the cohorts into two groups (cancer and non-cancer participants), there was a weak negative correlation between time and titer in the control group (r = −0.215; *p* = 0.002), while in the cancer group there was no association (*p* = 0.23).

## 4. Discussion

This study evaluated the efficacy of the BNT162b2 mRNA COVID-19 vaccine in patients with solid malignancies receiving active anti-neoplastic treatment. Our results indicate that cancer patients treated with anti-neoplastic therapy had a reduced humoral response following immunization with the full course of the vaccine, manifested by significantly more non- responders and lower antibody levels, compared with non-cancer individuals. Stratification of the patients according to the regimen, revealed that chemotherapy is the reason for the decreased vaccine immunogenicity. These novel results are in accordance with the known inhibitory effects of chemotherapy on the immune system, one of which is lymphocytopenia. However, cancer patients receiving treatments other than chemotherapy (immunotherapy, targeted therapy) had similar serologic response as participants without cancer.

To further validate these results, we performed a matched case-control analysis for the cancer and non-cancer cohorts, based on age [23], gender [24], and interval between the test and the vaccine [25]. The results according to this model were in the same line with the previous analysis, indicating significantly more cancer patients were seronegative. Moreover, this analysis confirmed the results in the chemotherapy and non-chemotherapy treated sub-groups. Likewise, two recently published articles described a reduced immune response to SARS-CoV-2 in patients with solid malignant neoplasms undergoing active cancer therapy [19,26]. Monin et al. have lately reported better immunogenicity after two versus one vaccine dose in cancer patients. However, the study population that received both vaccine doses was small (31 patients) and included hematological and solid cancers. Massarweh et al. [19] described a 90% seropositivity rate in 102 patients with solid cancers tested at least 12 days after receipt of the second BNT162b2 mRNA vaccine dose, compared with 100% seropositivity in non-cancer controls. In comparison with solid tumors, a significantly lower seroconversion rate was observed in patients with hematologic malignancies (85%), particularly recipients following highly immunosuppressive therapies such as anti-CD20 therapies [27].

Reduced antibody response was reported also in patients with solid tumors vaccinated against influenza and hepatitis [28,29,30,31]. Our results are consistent with those reported for immunosuppressed populations such as patients with chronic lymphocytic leukemia [14], and organ transplant recipients [32]. Immunosuppression has been shown to decrease the immune response in other vaccine studies, and particularly in patients with hematological malignancies who developed severely diminished antibody responses compared with healthy individuals [14,15,16]. These populations are at increased risk of SARS-CoV-2 infection despite vaccination.

One of the principal COVID-19 vaccines aim, reducing the probability of severe COVID-19 infection, is particularly relevant for cancer patients who are at an increased risk of developing a serious form of the disease. The minimum level of antibodies that result in protection from infection with SARS-CoV-2 is not known. The reduced sero-response may lead to lower vaccine efficacy or a shorter period of immunoprotection after vaccination. Moreover, it has been reported that the antibody titer decreases significantly within a short period after the 2nd dose of vaccine administration [33,34]. Consistent with this, a recent study in US Marine recruits assessed rates of COVID-19 re-infection in sero-positive participants after recovery and found that lower anti-spike IgG levels were associated with a higher risk of re-infection [35]. Interestingly, in the short interim time since collecting the study results, one of our seronegative chemotherapy treated cancer patients developed symptomatic COVID-19. Thus, the lower ability to mount an optimal immune response in chemotherapy treated cancer patients is an important factor that has to be considered, in addition to the vulnerability of these patients to SARS-CoV-2 infection, due to the effects of the malignant disease and the anti-neoplastic treatment. While the CDC, at this stage, does not recommend antibody testing after COVID-19 vaccines or re-vaccination in sero-negative individuals [12], we suggest that antibody testing may be suitable for vaccinated cancer patients receiving chemotherapy and that an additional boost dose(s), or a different vaccine composition might be considered. Nevertheless, since the significance of the IgG levels and sero-negativity is still unclear, further studies that will assess the risk for symptomatic COVID-19 in vaccinated cancer patients are needed. Besides serum titers, antigen specific CD4^+^ and CD8^+^ T cell responses also contribute to the COVID-19 vaccine efficacy [36]. In asymptomatic or mild COVID-19 SARS-CoV-2–specific memory T cells were demonstrated to be protective also in seronegative patients [37]. In accordance with this, a recent study (preprint only), reported T cell responses in the majority of vaccinated cancer patients, including those with low neutralizing antibody responses [38]. These data suggest that vaccination may protect, at least partially, cancer patients and reduce the likelihood of severe COVID-19. Comprehensive analysis of cellular immune responses to vaccination in patients with cancer and in other immunocompromised patients are warranted.

The recommendation of the American Society of Clinical Oncology (ASCO) is to vaccinate cancer patients that receive active anti-cancer treatment in between cycles of therapy and after appropriate waiting periods for patients receiving aggressive therapy with stem cell transplants and immune globulin treatment [13]. We did not include in the statistical analysis the timing of serologic tests relative to the chemotherapy schedule. The treatment schedules have a wide range of differences in the number of treatment days and the frequency of each cycle. Still, there is no information nowadays of a known effect of such an interval.

An additional novel insight of our study is the unimpaired humoral antibody response to the vaccine in cancer patients receiving non chemotherapy treatments, of which most were immunotherapy. Our results might be explained by the mechanism of action of checkpoint inhibitors that work on the immunological synapse of PD1-PDL1 or CTLA4 and thus accelerate antineoplastic activity of CD8 lymphocytes and are not considered to cause immunosuppression [39].

Limitations of this study are that other factors that may affect antibody levels following vaccination, were not included in our analysis, e.g., previous lines of systemic and radiation therapy, underlying diseases, preexisting immunity and genetic polymorphisms. The effect of anti-neoplastic regimens on cellular immunity was not measured in this study. Additionally, hematological malignancies were not part of the scope of this study. Lastly, the study was not powered to allow analysis according to cancer type, specific chemotherapy protocols or immunotherapy particular pathways.

## 5. Conclusions

Cancer patients vaccinated while undergoing chemotherapy treatment have a reduced humoral response to the BNT162b2 vaccine and might not be protected against SARS-CoV-2 infection. Immunogenicity induced by vaccination in cancer patients treated with regimens other than chemotherapy did not differ from that in non-cancer individuals. Based on our study results, it is reasonable to consider re-evaluating the recommendations for vaccinated cancer patients treated with chemotherapy. We suggest periodic antibody assessment after COVID-19 vaccination, continuation of precautionary measures such as social distancing and facial masking in seronegative patients and the potential addition of a vaccine boost dose(s).

## Figures and Tables

**Table 1:** Participants’ characteristics.

Characteristic	Cancer Participants	Non-Cancer Participants	Statistical Test	*p* *
Number (*n*)	140	215		
Age, mean (SD) [years]	65.3 (1.4)	62.5 (13)	*t*	0.05
Male [%]	76 (54%)	80 (37.2%)	χ^2^	0.002
Time from second vaccine to test, median (IQR) [days]	35 (23.5–45)	51 (43–62)	Mann-Whitney	<0.001
Type of Cancer, *n* (%)				
Gastrointestinal	48 (34.2%)			
Breast	30 (21.4%)			
Lung	27 (19.3%)			
Urinary	13 (9.3%)			
Gynecological	9 (6.4%)			
Other types	13 (9.3%)			
Treatment, *n* (%)				
Chemotherapy	73 (52%)			
Non-chemotherapy	67 (48%)			
Immunotherapy	43 (30.7%)			
Treatment type, *n* (%)				
Metastatic	109 (77.8%)			
Adjuvant	23 (16.4%)			
Neoadjuvant	8 (5.7%)			

* *p* < 0.05 is considered statistically significant.

**Table 2:** SARS-CoV-2 mRNA Vaccine Response after the second dose.

Group	Analysis	Parameter	Cancer Participants	Non-Cancer Participants	Statistical Test	*p*
All Participants	Unmatched	Number (*n*)	140	215		
		Seronegative, *n* (%)	20 (14.3)	3 (1.4)	Fisher	<0.001
		IgG [AU], median (IQR)	2231 (445–8023)	4100 (2231–6774)	Mann-Whitney	0.001
	Matched * 14 d	Number (*n*)	99	99		
		Seronegative, *n* (%)	16 (16.2)	3 (3)	McNemar	0.004
		IgG [AU], median (IQR)	1771 (284–7292)	2993 (1775–6002)	Wilcoxon	0.52
	Matched * 7 d	Number (*n*)	82	82		
		Seronegative, *n* (%)	11 (13.4)	3 (3.7)	McNemar	0.057
		IgG [AU], median (IQR)	1918 (414–7176)	3677 (1842–6777)	Wilcoxon	0.176
Chemo Treated vs. Non-Cancer	Unmatched	Number (*n*)	73	215		
		Seronegative, n (%)	17 (23.3)	3 (1.4)	Fisher	<0.001
		IgG [AU], median (IQR)	1361 (162–4030)	4100 (2231–6774)	Mann-Whitney	<0.001
	Matched * 14 d	Number (*n*)	57	57		
		Seronegative, *n* (%)	13 (22.8)	2 (3.5)	McNemar	0.007
		IgG [AU], median (IQR)	821 (162–4177)	3559 (1691–5957)	Wilcoxon	0.035
	Matched * 7 d	Number (*n*)	45	45		
		Seronegative, *n* (%)	8 (17.7)	1 (2.2)	McNemar	0.039
		IgG [AU], median (IQR)	864 (256–4178)	3329 (1691–7491)	Wilcoxon	0.013
	Unmatched	Number (*n*)	67	215		
		Seronegative, *n* (%)	3 (4.5)	3 (1.4)	Fisher	0.12
		IgG [AU], median (IQR)	5088 (890–14925)	4100 (2231–6774)	Mann-Whitney	0.627
		Number (*n*)	42	42		
		Seronegative, *n* (%)	3 (7.1)	1 (2.4)	McNemar	0.625
		IgG [AU], median (IQR)	3168 (573–10465)	2977 (1920–6860)	Wilcoxon	0.163
	Matched * 7 d	Number (*n*)	37	37		
		Seronegative, *n* (%)	3 (8.1)	2 (5.4)	McNemar	>0.99
		IgG [AU], median (IQR)	3619 (851–10315)	4792 (2117–6926)	Wilcoxon	0.57
Immuno Treated vs. Non-Cancer	Unmatched	Number (*n*)	43	215		
		Seronegative, *n* (%)	3 (7)	3 (1.4)	Fisher	0.06
		IgG [AU], median (IQR)	2714 (611–15564)	4100 (2231–6774)	Mann-Whitney	0.6
	Matched * 14 d	Number (*n*)	26	26		
		Seronegative, *n* (%)	3 (11.5)	1 (3.8)	McNemar	0.625
		IgG [AU], median (IQR)	2136 (443–8126)	3066 (2014–7772)	Wilcoxon	0.97
	Matched * 7 d	Number (*n*)	21	21		
		Seronegative, *n* (%)	3 (14.3)	2 (9.5)	McNemar	>0.99
		IgG [AU], median (IQR)	1771 (434–7535)	4066 (2081–6574)	Wilcoxon	0.715

Abbreviations: AU, arbitrary antibody units; IQR, interquartile range. * The groups were matched according to age (±3 years), gender and days from vaccination (within 14 or 7 days of each other).

## Data Availability

The data presented in this study are available on request from the corresponding authors.

## Supplementary Materials

The following are available online at https://www.mdpi.com/article/10.3390/cancers13164191/s1, Figure S1: SARS-CoV-2 IgG antibodies titer. Table S1: SARS-CoV 2 IgG Titer Values by Cancer Type. Table S2: Participants’ Characteristics stratified according to three IgG antibodies categories. Table S3: SARS-CoV-2 mRNA Vaccine Response after the second dose among chemotherapy and non-chemotherapy treated cancer patients.
