# Supplementary material for: Efficacy of the mRNA-Based BNT162b2 COVID-19 Vaccine in Patients with Solid Malignancies Treated with Anti-Neoplastic Drugs
Source: Cancers (Basel). 2021 Aug 20;13(16):4191. doi: 10.3390/cancers13164191 (PMC8391288; doi:10.3390/cancers13164191)
Supplement: Supplementary file 1 [file cancers-13-04191-s001.zip › cancers-1273021-supplementary.pdf]

cancers

MDPI

*Article*

# Efficacy of the mRNA-Based BNT162b2 COVID-19 Vaccine in Patients with Solid Malignancies Treated with Anti-Neoplastic Drugs

**Abed Agbarya, Ina Sarel, Tomer Ziv-Baran, Sivan Agranat, Orna Schwartz, Ayelet Shai, Sharon Nordheimer, Shlomit Fenig, Yelena Shechtman, Ella Kozlener, Tarek Taha, Haitam Nasrallah, Roma Parikh, Nadav Elkoshi, Carmit Levy, Rasha Khoury and Ronen Brenner**

**Supplementary Materials**

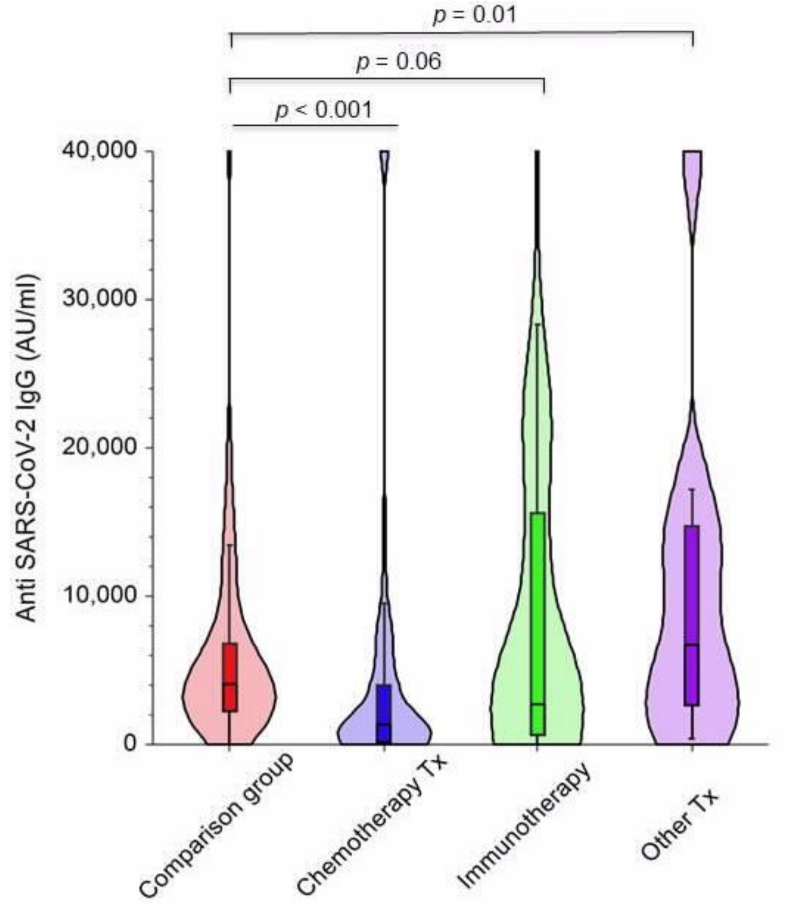


**Figure S1.** SARS-CoV-2 IgG antibodies titer**.** Violin graphs and box plots of the distribution of anti-SARS-CoV-2 antibody levels (AU/ml). The median and quartiles are presented as horizontal lines within the box plot. Chemotherapy treated and comparison non-cancer groups (unmatched). The median antibody levels are significantly lower in the chemotherapy treated group, 1361 AU/ml vs. 4100 AU/ml in the comparison group, ($p<0.001$). The lower IgG values, as apparent from the wider bottom distribution, are more frequent in this group. The Immunotherapy and Other treatment group antibody levels, were not significantly different than the non-cancer comparison group (p=0.06, p=0.01), respectively.

**Table S1.** SARS-CoV 2 IgG Titer Values by Cancer Type.

| | | Cancer Participants (*n* = 140) | | | |
|---|---|---|---|---|---|
| **Cancer type** | **Number (n)** | **Seronegative (<50 AU/ml) n (%)** | **Borderline (50–150 AU/ml) n (%)** | **Seropositive (>150 AU/ml) n (%)** | **IgG, mean (IQR)** |
| GI | 41 | 4 (9.8) | 3 (7.3) | 34 (82.9) | 4343 (217–5532) |
| Lung | 27 | 2 (7.4) | 0 | 25 (92.6) | 8029 (570–10598) |
| Breast | 30 | 4 (13.3) | 1 (3.3) | 25 (83.3) | 8387 (888–13132) |
| Pancreas | 7 | 2 (28.6) | 1 (14.3) | 4 (57.1) | 1494 (44–1742) |
| Urinary | 13 | 1 (7.7) | 0 | 12 (92.3) | 3524 (538–5426) |
| Sarcoma | 1 | 1 (100) | 0 | 0 | 0.7 |
| Head & Neck | 2 | 0 | 1 (50) | 1 (50) | 1721 |
| Melanoma | 7 | 0 | 0 | 7 (100) | 10708 (1489–20791) |
| Skin-non Melanoma | 3 | 0 | 0 | 3 (100) | 2622 |
| Gynecological | 9 | 0 | 0 | 9 (100) | 10777 (2448–16564) |

**Table S2.** Participants' Characteristics stratified according to three IgG antibodies categories.

| | Cancer Patients | | | | Non-Cancer Participants | | | |
|---|---|---|---|---|---|---|---|---|
| **Characteristic** | **All** | **Seronegative (<50 AU/ml)** | **Borderline (50–150 AU/ml)** | **Seropositive (>150 AU/ml)** | **All** | **Seronegative (<50 AU/ml)** | **Borderline (50–150 AU/ml)** | **Seropositive (>150 AU/ml)** |
| Number (n) | 140 | 14 | 6 | 120 | 215 | 1 | 2 | 212 |
| Age, mean years (SD) | 65.3 (13.4) | 66.8 (19.2) | 66 (13.3) | 65 (12.8) | 62.5 (13) | 69 | 67,82 | 62.7 (12.9) |
| Male, (%) | 76 (54%) | 8 (57) | 4 (66.6) | 64(53.3) | 80 (37.2) | 1 | 2 (100) | 77 (36.3) |
| Time from 2nd COVID-19 vaccine, median days (IQR) | 35 (23.5–45) | 35 (33–36) | 35 (25–40) | 36 (23–47) | 51 (43–62) | 62 | 39,28 | 51 (43–62) |
| Type of Cancer, n (%) | | | | | | | | |
| Gastrointestinal | 48 (34.2) | 6 | 4 | 38 | | | | |
| Breast | 30 (21.4) | 4 | 1 | 25 | | | | |
| Lung | 27 (19.3) | 2 | 0 | 25 | | | | |
| Urinary | 13 (9.3) | 1 | 0 | 12 | | | | |
| Gynecological | 9 (6.4) | 0 | 0 | 9 | | | | |
| Other types | 13 (9.3) | 1 | 1 | 11 | | | | |
| Treatment, n (%) | | | | | | | | |
| Chemotherapy | 73 (52) | 12 | 4 | 57 | | | | |
| Not Chemotherapy | 67 (48) | 2 | 2 | 63 | | | | |
| Immunotherapy | 43 (30.7) | 1 | 2 | 40 | | | | |
| Treatment type, n (%) | | | | | | | | |
| Metastatic Treatment | 109 | 8 | 5 | 96 | | | | |
| Adjuvant Treatment | 23 | 2 | 0 | 21 | | | | |
| Neo-Adjuvant Treatment | 8 | 4 | 1 | 3 | | | | |

**Table S3.** SARS-CoV-2 mRNA Vaccine Response after the second dose among chemotherapy and non-chemotherapy treated cancer patients.

| Characteristic | Chemotherapy Treated Patients | Non-Chemotherapy Treated Patients | Statistical Test | *p* |
|---|---|---|---|---|
| Number (n) | 73 | 67 | | |
| Time from 2nd COVID-19 vaccine, mean days (min–max) | 35.8 (9–72) | 34.3 (7–64) | Mann-Whitney | 0.66 |
| Seronegative , n(%) | 17 (23.3) | 3 (4.5) | Fisher | 0 |
| IgG, median (IQR) | 1361 (162.4–4030) | 5088 (890–14925) | Mann-Whitney | <0.001 |
